# Multifunctional Properties of Polyhedral Oligomeric Silsesquioxanes (POSS)-Based Epoxy Nanocomposites

**DOI:** 10.3390/polym15102297

**Published:** 2023-05-13

**Authors:** Liberata Guadagno, Andrea Sorrentino, Raffaele Longo, Marialuigia Raimondo

**Affiliations:** 1 Department of Industrial Engineering, University of Salerno, Via Giovanni Paolo II, 132, 84084 Fisciano, Italy; lguadagno@unisa.it (L.G.); rlongo@unisa.it (R.L.); 2 Institute for Polymers, Composites, and Biomaterials (IPCB-CNR), Via Previati n. 1/E, 23900 Lecco, Italy; andrea.sorrentino@cnr.it

**Keywords:** polyhedral oligomeric silsesquioxane (POSS), thermosetting resins, multifunctional materials, thermal properties, tunneling atomic force microscopy (TUNA) technique, structural composites, mechanical properties

## Abstract

In this study, a tetrafunctional epoxy resin was loaded with 5 wt% of three different types of polyhedral oligomeric silsesquioxane (POSS) compounds, namely, DodecaPhenyl POSS (DPHPOSS), Epoxycyclohexyl POSS (ECPOSS), Glycidyl POSS (GPOSS), and 0.5 wt% of multi-walled carbon nanotubes (CNTs) in order to formulate multifunctional structural nanocomposites tailored for aeronautic and aerospace applications. This work aims to demonstrate how the skillful combination of desired properties, such as good electrical, flame-retardant, mechanical, and thermal properties, is obtainable thanks to the advantages connected with nanoscale incorporations of nanosized CNTs with POSS. The special hydrogen bonding-based intermolecular interactions between the nanofillers have proved to be strategic in imparting multifunctionality to the nanohybrids. All multifunctional formulations are characterized by a Tg centered at values close to 260 °C, fully satisfying structural requirements. Infrared spectroscopy and thermal analysis confirm the presence of a cross-linked structure characterized by a high curing degree of up to 94% and high thermal stability. Tunneling atomic force microscopy (TUNA) allows to detect the map of the electrical pathways at the nanoscale of the multifunctional samples, highlighting a good dispersion of the carbon nanotubes within the epoxy resin. The combined action of POSS with CNTs has allowed to obtain the highest values of self-healing efficiency if compared to those measured for samples containing only POSS in the absence of CNTs.

## 1. Introduction

Composite materials nowadays represent a very widespread class of materials because they find a wide range of applications in the real world, thanks also to their versatility which makes them suitable even for very particular and specific applications [1,2,3]. Polymer-based nanocomposite materials are showing rapid and promising development. In the last ten years, material scientists have directed considerable interest, both academic and industrial, to hybrid organic–inorganic composite materials thanks to their peculiar characteristics that arise from the synergies between the properties of their components. More precisely, the aim of obtaining hybrid organic–inorganic materials consists in improving the final properties compared to the properties of their counterparts [4,5,6,7,8] while reducing their particular limits. Such materials can be suitably developed by exploiting the synergistic effect deriving from the combination of components whose properties have already been investigated for specific applications but which provide an additional dimension to their properties by becoming part of the hybrid compound [9,10]. Through this synergy of properties, it is possible to create multifunctional materials that can offer new solutions that fully meet the requirements of a targeted application [11,12,13,14]. These materials have exhibited dramatic variations in properties ranging from impressive improvements in mechanical properties to excellent electrical properties [15,16] with profound implications in structure–property relationships at the nanoscale [17], leading to a growing interest in the area of nanocomposites. Compared to those based on micro composites, composites based on nanofillers offer advantages deriving from the greater interfacial contact area among the components in the nanocomposites, allowing more effective interactions between the intermediate phases [18,19]. However, the increased surface area of the nanoparticles also entails drawbacks related to complex preparation procedures, such as controlled mixing/composition and stabilization of the resulting mixture, which are necessary to obtain effective dispersion of the nanofiller initially randomly dispersed in the polymer matrix [20,21]. Additionally, the interactions between nanofillers within the polymer blend are stronger, increasing strength, thermal stability, and other useful properties for composite applications [22]. Essential aspects in forming these systems are the uniformity, the size of the domains, and the compatibility of the different components, the factors that directly influence all the properties as a whole.

In this regard, it can be noted that while the mechanisms of incorporation of inorganic reinforcements into organic polymers on the micrometric scale are certainly known and well-studied [23], the reduction in the dimensions of the inorganic phase to the nanometric scale [24] constitutes a new scientific horizon which will undoubtedly require further studies and insights. Stimulated by the enthusiasm that has exploded around nanotechnologies [25], polymeric matrix nanocomposite materials are the category that is showing the fastest and most promising development among the others. They consist of a polymeric matrix, such as epoxy, polystyrene, polyaniline, polyurethane, poly (vinylidene fluoride), Nafion, poly-carbonate, and poly (ethylene terephthalate) [21], modified by the addition of particles characterized by at least one nanometric size. In this work, as a matrix for the formulated composites, an epoxy resin was used, which, among thermosets, is the preferred one due to its high modulus, low shrinkage, exceptional chemical and corrosion resistance, as well as good adhesion properties. However, the use of epoxies is limited due to their fragile nature. Hence, to improve the properties of epoxies without compromising their superior characteristics, carbon nanotubes, which act as a nano reinforcement [26,27,28,29,30,31,32,33,34] due to their extraordinary physical and mechanical properties, as well as high aspect ratio [35,36], have been added.

Particularly critical in the design of hybrid organic–inorganic systems is the control of the intimate mixing between the different phases [37]. In this regard, the tailoring of polymer–nanomaterial interaction is of fundamental importance and represents a challenge involved in preparing nanocomposites. To formulate high-performance materials for several potential application areas (aerospace, automotive, defense, biomedical, civil engineering, food packaging, textile, telecommunications) [38,39,40,41,42,43,44,45], great efforts are focusing on epoxy-based nanocomposites reinforced with well-defined nanostructured compounds.

In this context, the POSS cages to be incorporated into polymeric matrices are becoming increasingly important. They represent an innovative class of materials with a unique nanometric structure [46,47,48] and awesome mechanical and thermal properties that make them ideal reinforcing reagents [49,50,51,52,53,54,55,56,57,58,59].

POSS have at least two completely unique properties: (a) their chemical composition (RSiO_1.5_) is a hybrid, intermediate between that of silica (SiO_2_) and that of silicone (R_2_SiO); (b) POSS compounds are “large” (from 1 to 3 nm) and similar in size to most of the statistical balls made up of long macromolecular chains [60].

This work demonstrates that the interactions between CNT and POSS compounds affect the final performance of the formulated hybrid materials.

Dynamic mechanical analysis (DMA) shows that, among the multifunctional nanocomposites, the highest storage modulus values over the whole investigated temperature range were recorded for the epoxy sample loaded with CNTs and GPOSS. This sample shows the same trend shown by the sample containing only carbon nanotubes in the absence of GPOSS. Furthermore, all multifunctional composites show an electrical conductivity of 10^−3^ S/m, which is a very satisfactory value to counter the electrical insulating property of epoxy systems.

TUNA analysis, employed in this work, is a powerful investigation tool that allows obtaining a correlation between the electrical properties and the morphological characteristics of conductive materials within which it is possible to detect electrical pathways at micrometric/nanometric level originating from the nanocharge dispersed in the insulating matrix [61,62,63,64,65,66,67]. For the first time, TUNA was used to compare conductive networks detectable for the three different POSS-based nano-filled epoxy resins. This comparison allows obtaining information on the local electrical properties of nanodomains and morphological features of the conductive CNTs as well as on the effectiveness of their dispersion in the epoxy matrix responsible for improving the performance of the formulated materials. In this way, it is possible to highlight the advantages offered by the synergistic combination of conductive carbon nanotubes with POSS compounds to give rise to hybrid nanocomposites with superior performance suitable for advanced applications in aeronautics and aerospace.

## 2. Materials and Methods

In this work, multi-walled carbon nanotubes (3100 Grade—Nanocyl S.A, Sambreville, Belgium), here labeled as CNT, were used as conductive nanofillers. The morphological and structural parameters of CNT have been investigated in our previous paper [68].

More detailed information on the CNT is contained in Supplementary Materials. The unfilled epoxy matrix, where the resin tetraglycidyl methylene dianiline (TGMDA) (80 wt%) was mixed with the reactive diluent1,4-Butanediol diglycidyl ether (BDE) (20 wt%), and a stoichiometric amount of curing agent diaminodiphenyl sulfone (DDS) was solubilized at 120 °C for 20 min is indicated with the abbreviation EP. All these products were bought at Sigma-Aldrich. GPOSS functionalized with glycidyl groups, ECPOSS functionalized with epoxycyclohexyl groups, and DPHPOSS functionalized with phenyl groups were bought at Hybrid Plastics Company (Hattiesburg, MS, USA). Figure S1 shows the chemical structures of GPOSS, ECPOSS, DPHPOSS, and information about the characteristics and aspects of the investigated POSS compounds are reported in Supplementary Materials.

The detailed procedure by which the samples EP, EP+0.5%CNT, EP+5%GPOSS+0.5%CNT, EP+5%DPHPOSS+0.5%CNT, and EP+5%ECPOSS+0.5%CNT were prepared is reported in Supplementary Materials. They were subjected to a heat treatment in an oven at 125 °C for 1 h and then at 200 °C for 3 h, which led to their solidification.

In this work, various experimental techniques have been used to carry out the characterization of the formulated materials. Detailed technical information on Dynamic Mechanical Analysis (DMA), Fourier Transform Infrared Spectroscopy (FTIR), Differential Scanning Calorimetry (DSC), Thermogravimetric analysis (TGA), Field Emission Scanning Electron Microscopy (FESEM), self-healing efficiency evaluation, and electrical characterization by TUNA has been provided in Supplementary Materials.

## 3. Results and Discussion

### 3.1. Dynamic Mechanical Behavior of the Epoxy Samples

#### DMA Storage Modulus and Loss Factor (tanδ) Curves of the Epoxy Samples

**Figure 1:**
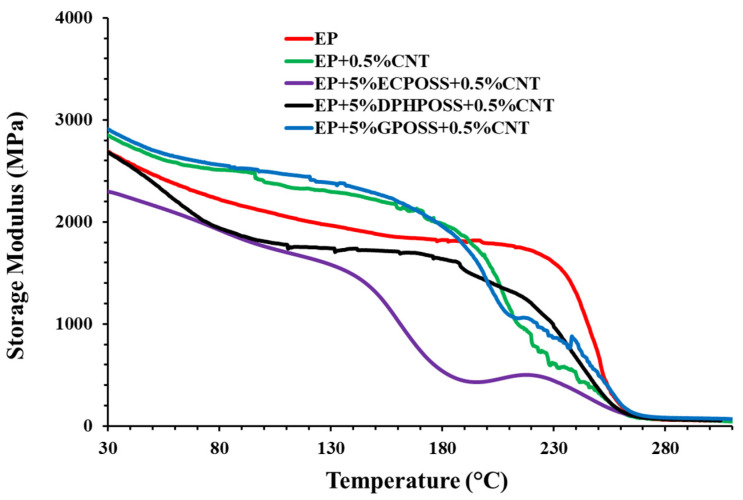
Storage modulus of the cured epoxy samples.

Figure 1 shows the storage modulus of the cured epoxy samples EP, EP+0.5%CNT, EP+5%ECPOSS+0.5%CNT, EP+5%DPHPOSS+0.5%CNT, and EP+5%GPOSS+0.5%CNT. The temperature range investigated is between 30 °C and 310 °C.

**Figure 2:**
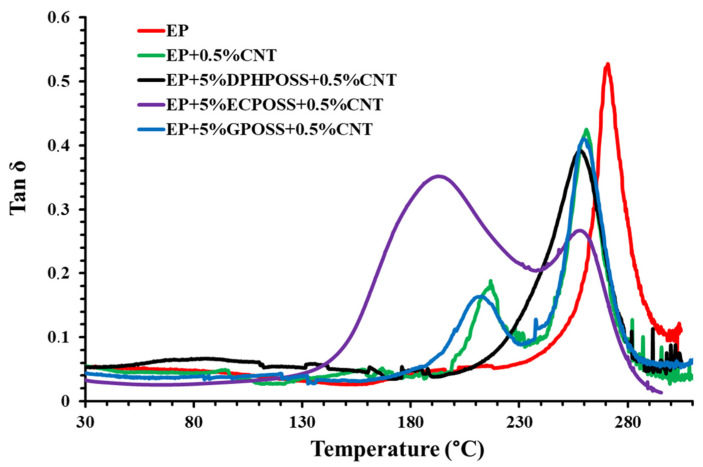
Loss factor (tanδ) of the cured epoxy samples.

To evaluate the influence of POSS and carbon nanotubes on the dynamic–mechanical properties of these multifunctional nanocomposites, a comparison was also made considering the unfilled resin EP and the sample loaded with carbon nanotubes EP+0.5%CNT. We can easily see that carbon nanotubes alone, or in combination with GPOSS, in the hosting matrix exert a reinforcing effect (see green curve) compared to the unfilled epoxy matrix (see red curve), as can be seen from the increase in the storage modulus throughout the temperature range from 30 °C up to about 190 °C, to then register a drop. This growth is attributable to the intense interfacial interactions between conductive nanofiller and matrix. In fact, it was demonstrated that the increase in the storage modulus is not connected with the presence of GPOSS within the epoxy system [32]. However, the presence of GPOSS, which is functionalized with glycidyl groups, makes it compatible with the epoxy precursors in which it was found to be completely soluble [49], unlike ECPOSS and DPHPOSS. The complete solubility of GPOSS in the hosting liquid epoxy precursors and the presence of oxirane rings in the structure of GPOSS allow the formation of a continuous crosslinking network in the epoxy matrix. This allows obtaining a nanocomposite manifesting a trend of the storage modulus similar to that of the sample containing only the CNTs. Thus, among multifunctional nanocomposites, the highest storage modulus values were recorded for the sample EP+5%GPOSS+0.5%CNT (see the light blue curve), in which carbon nanotubes are combined with GPOSS. For the sample EP+5%DPHPOSS+0.5%CNT containing DPHPOSS and CNT (black curve), a lowering of the storage modulus both compared to the unfilled resin EP and samples EP+0.5%CNT and EP+5%GPOSS+0.5%CNT was recorded in the temperature range between approximately 30 °C and 190 °C. This decrease is most likely due to tiny DPHPOSS aggregates within the resin since DPHPOSS is functionalized with phenyl groups that impede its complete solubilization in the matrix. It has been demonstrated that DPHPOSS, through two steps of ultrasonication and magnetic stirring at 120 °C, can be well dispersed in the epoxy mixture leaving tiny aggregates anyway [49]. Among the multifunctional nanocomposites, the lowest storage modulus values were recorded for the sample EP+5%ECPOSS+0.5%CNT (see purple curve) containing ECPOSS and CNT. Although ECPOSS showed a good dispersion in the liquid resin, the presence of very few randomly distributed aggregates in the resin [49] led to inhomogeneities in the sample with the formation of zones with a different chemical composition of the cross-linking network, as detectable in Figure 2, where the profile of the loss factor (tanδ) curves of the cured epoxy samples EP, EP+0.5%CNT, EP+5%ECPOSS+0.5%CNT, EP+5%DPHPOSS+0.5%CNT, EP+5%GPOSS+0.5%CNT is shown.

Observing samples EP+0.5%CNT and EP+5%GPOSS+0.5%CNT, we can state that the lower temperature transition in the profile of the curve related to the loss factor does not depend on the presence of GPOSS, as already observed for the storage modulus. For the sample with ECPOSS, the transition at a lower temperature is more intense and broadened; this allows the sample to develop toughness due to the increased side-chain motion. With a further increase in temperature, the alpha transition (Tα), also called the glass transition (Tg), is observed. Therefore, the profile of the curve related to the loss factor clearly indicates the existence of a phase where the segments of the chain are endowed with greater mobility. The lowest temperature transition occurs due to the local motion of the polymer chains as opposed to the large-scale cooperative motion that accompanies the Tg. For the sample EP+5%DPHPOSS+0.5%CNT, we can observe the tanδ peak (Tg) at 260 °C. However, all multifunctional formulations have a Tg centered at values close to 260 °C, which fully satisfies structural requirements. In conclusion, the presence of carbon nanotubes affects the structure of the epoxy matrix since, during the solidification phase of the sample, it can cause discontinuities in the crosslinking reaction giving rise to phases with a lower Tg. This explains the presence in the formulations containing CNT, CNT and ECPOSS, CNT and GPOSS of a second peak due to a further phase having a different crosslinking density.

### 3.2. FTIR Investigation of the Epoxy Samples

#### Curing Behavior Evaluation of the Epoxy Samples before and after Curing Treatment

**Figure 3:**
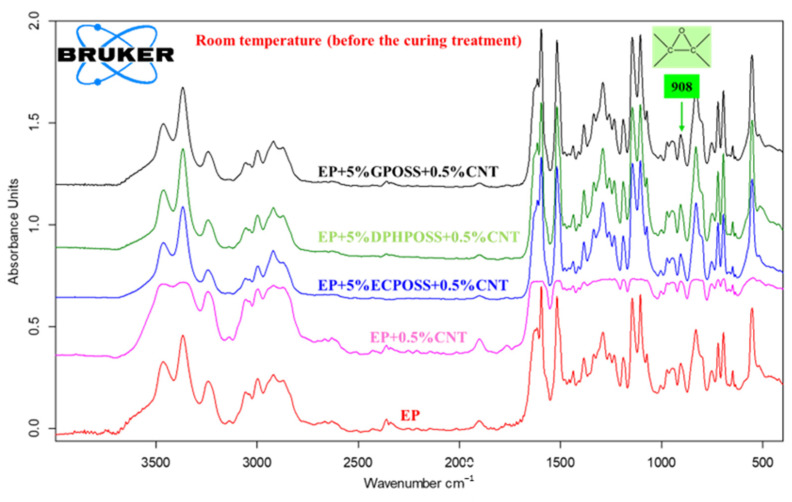
FTIR spectra of epoxy samples at room temperature (before the curing treatment).

**Figure 4:**
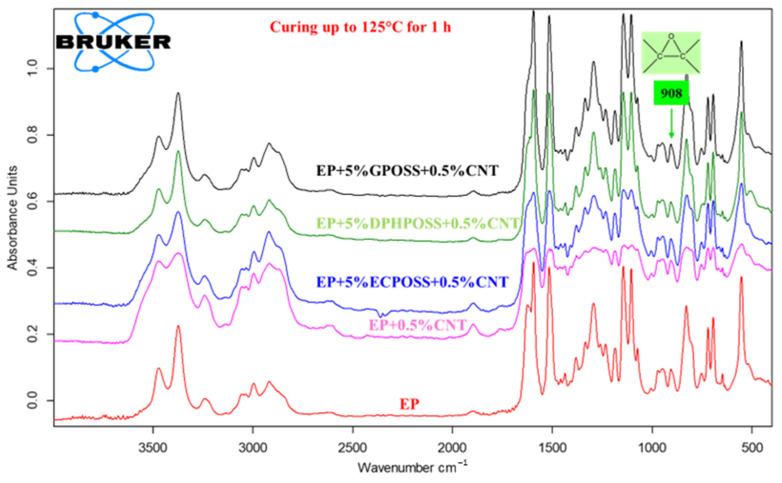
FTIR spectra of epoxy samples after curing up to 125 °C for 1 h.

**Figure 5:**
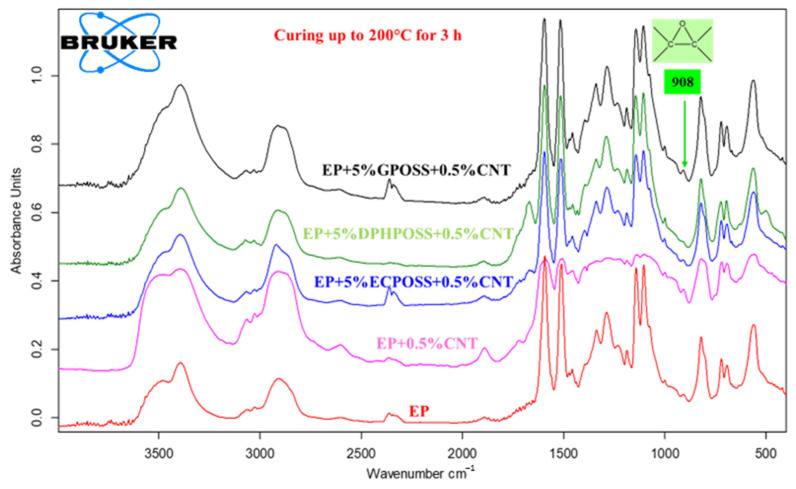
FTIR spectra of epoxy samples after curing up to 200 °C for 3 h.

In this work, the curing degree of epoxy systems was evaluated by infrared spectroscopy, which allows one to follow the evolution of the curing process and obtain molecular-level information useful for a better understanding of the structure–property relationships of the materials. It is worth noting that the crosslinking kinetics provide information on the creation of the three-dimensional (3D) network of an epoxy formulation due to the reaction of a suitable hardener agent (in our case, DDS, which is a primary aromatic amine) with the epoxy rings (in our case, epoxy precursors represented by the tetrafunctional resin TGMDA and the reactive diluent BDE), which are located at the ends of the chains and which act as reactive sites for the crosslinking reaction. Curing kinetics play a decisive role in the final performance of epoxy systems, and furthermore, the time required for the curing of an epoxy system is of considerable interest in industrial applications. If the final material has an anomaly in performance, it could be due to the incomplete crosslinking reaction. The curing degree of multifunctional hybrid systems evaluated through spectroscopic analysis also allows us to understand their thermal and mechanical behavior. The FTIR investigation, which reproduces the steps of the curing cycle carried out in the oven, demonstrates the effectiveness of the curing process to which the samples have been subjected. Figure 3, Figure 4 and Figure 5 show the FTIR spectra of epoxy samples EP, EP+0.5%CNT, EP+5%ECPOSS+0.5%CNT, EP+5%DPHPOSS+0.5%CNT, and EP+5%GPOSS+0.5%CNT at room temperature (before the curing treatment), after curing up to 125 °C for 1 h and after curing up to 200 °C for 3 h, respectively. FTIR spectroscopy has been used to investigate the curing behavior of epoxy resin by determining the band intensity change of functional groups before and after curing reactions of epoxy resin and also during the reaction time at a given temperature. Along with the band of the epoxy functional group, which is the characteristic group in the epoxy resin, the primary amine is the most useful band in the monitoring curing process due to the absence of overlapping. In particular, in this work, we have followed the change in the absorption intensity of the FTIR peak at 908 cm^−1^ associated with the epoxy group responsible for the polymerization reaction. This peak is considered the maximum for uncured epoxy resins, decreasing significantly at higher temperatures and reaction times. In this way, from the direct comparison between the different FTIR spectra of the epoxy samples, before and after curing, we can examine the progress of the curing process as it passes from room temperature up to 125 °C and then to 200 °C for the defined reaction times, thus obtaining useful information about the chemical reactions that lead to the formation of a highly crosslinked 3D network. In this work, the curing mechanism of TGMDA epoxy resin in the presence of the curing agent DDS (primary amine) was evaluated by FTIR. The amino group shows well-defined absorption bands. Although the N-H stretching is located between 3500 and 3300 cm^−1^, primary amines show a doublet (reflecting the symmetric and antisymmetric stretching modes), while the secondary amines show one single band [31]. We can easily observe that, for all formulations tested, the peak at 908 cm^−1^ is marked at room temperature before the curing process begins (see Figure 3). At a temperature of 125 °C after 1 h (see Figure 4), the peak, although reduced in intensity, is still evident and then disappears almost completely when the temperature is brought to 200 °C after 3 h (see Figure 5). The abatement of the peak at 908 cm^−1^ indicates that the curing process was found to be effective, leading to the formation of a highly crosslinked network. The effectiveness of the crosslinking reaction is also demonstrated by the disappearance of the N-H stretching vibration bands of DDS at 3060–3500 cm^−1^ band and the increase in the absorption bands of the O-H stretch (due to the attack of NH_2_ to the epoxide ring) at 3200–3650 cm^−1^. It was possible to appreciate the variation of the intensity of the peaks discussed above, taking as reference the following peaks, which do not change during the curing process: (a) strong asymmetric and symmetric SO_2_ stretching of the DDS hardener at 1143 cm^−1^ and 1105 cm^−1^, respectively; and (b) phenyl groups at 1595 cm^−1^ and 1515 cm^−1^ [31].

### 3.3. Thermal Behavior of the Epoxy Samples

#### 3.3.1. Differential Scanning Calorimetry (DSC) Analysis

**Figure 6:**
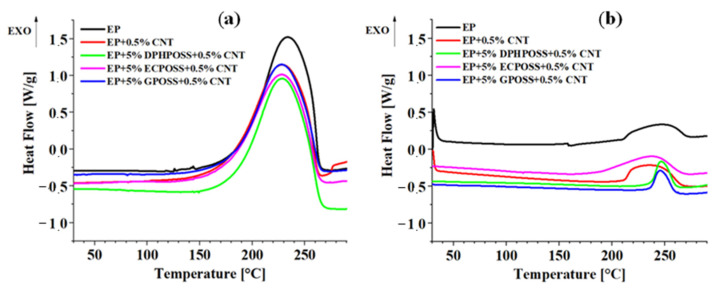
(**a**) Calorimetric curves (dynamic regime) of the epoxy samples. (**b**) Calorimetric curves (isothermal regime) of the epoxy samples.

Figure 6 shows the calorimetric curves of the epoxy samples EP, EP+0.5%CNT, EP+0.5%DPHPOSS+0.5%CNT, EP+0.5%ECPOSS+0.5%CNT, and EP+0.5%GPOSS+0.5%CNT solidified under dynamic (see graph on the left) and isothermal (see graph on the right) heating conditions (see experimental details in Supplementary Materials), respectively. Table 1 shows the values of the total heat of the reaction (ΔH_T_), the residual heat of the reaction (ΔH_Res_), and the percentage of the degree of cure (DC) for the epoxy samples. The DC was calculated from the total heat of reaction (ΔH_T_) of the first run curing reaction and the residual heat of reaction (ΔH_resid_) of the partially cured epoxy formulations. Detailed information on how the DC values were calculated is given in Supplementary Materials. The DC value of the unfilled resin EP is equal to 93%, while the sample EP+0.5%CNT, loaded with only carbon nanotubes CNT without POSS, shows a DC value of 89%. Among the multifunctional nanocomposites, we can observe that the highest DC value equal to 94% was recorded for sample EP+5%DPHPOSS+0.5%CNT, containing carbon nanotubes CNT in combination with DPHPOSS, while for sample EP+5%GPOSS+0.5%CNT, containing carbon nanotubes CNT in combination with GPOSS, there is a DC value equal to 91% and for sample EP+5%ECPOSS+0.5%CNT, containing carbon nanotubes CNT in combination with ECPOSS, a DC value equal to 86% was measured. The calorimetric data of Table 1 confirm the mechanical behavior of the analyzed epoxy formulations. In fact, the lowest DC values with respect to the DC value shown by the unfilled resin EP were detected for samples EP+0.5%CNT, EP+5%ECPOSS+0.5%CNT, and EP+5%GPOSS+0.5%CNT for which, as previously discussed, in the profile of loss factor (tanδ) (see Figure 2), we can observe the presence of a second peak due to a further phase having a different crosslinking density originated from the addition of the carbon nanotubes inside the resin.

#### 3.3.2. Thermogravimetric Analysis (TGA)

**Figure 7:**
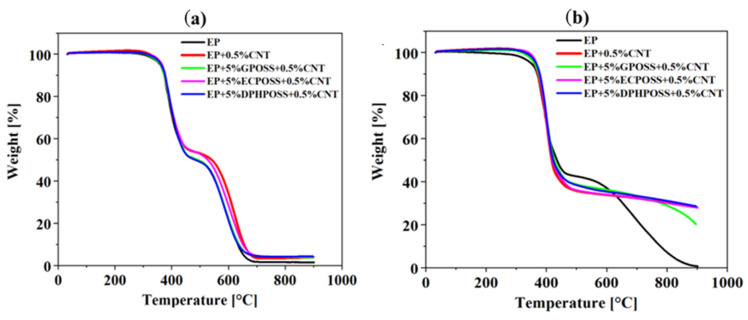
(**a**) TGA curves in air of the cured epoxy samples. (**b**) TGA curves in nitrogen of the cured epoxy samples.

Figure 7 shows the TGA curves in the air (see graph on the left) and in an inert (N_2_) atmosphere (see graph on the right) of the samples EP, EP+0.5%CNT, EP+5%GPOSS+0.5%CNT, EP+5%ECPOSS+0.5%CNT, and EP+5%DPHPOSS+0.5%CNT, respectively. Table 2 shows the values of thermal degradation temperatures corresponding to a weight loss of 5 wt% and 10 wt% (T_d5%_ and T_d10%_, respectively) and the values of residue at 900 °C, both in airflow and in nitrogen flow. In the air, within the experimental temperature range, we can observe a two-step thermal degradation process for the investigated samples, suggesting that the addition of POSS compounds and carbon nanotubes in the matrix does not considerably change the degradation mechanism of the formulation. In the air, a residual yield of 1.44 wt% was obtained at 900 °C for the unfilled resin EP (see Table 2). In the air, for the samples EP+0.5%CNT, EP+5%DPHPOSS+0.5%CNT, EP+5%ECPOSS+0.5%CNT, EP+5%GPOSS+0.5%CNT, the residual yield at 900 °C are as follows, respectively: 3.97 wt%; 4.31 wt%; 4.15 wt%; 3.65 wt%. For the samples EP+5%GPOSS+0.5%CNT and EP+5%DPHPOSS+0.5%CNT (green and blue curve, respectively), a thermostability similar to that shown by the unfilled resin EP (black curve) was observed. Instead, an increase in thermostability with respect to the EP resin was observed for the EP+0.5%CNT and EP+5%ECPOSS+0.5%CNT samples (red and fuchsia curve, respectively), especially in the second stage of the degradation process. In particular, in N_2_, in the first stage of the degradation, all the investigated formulations exhibit a substantial increase in thermostability compared to the unfilled resin EP, whereas the second step is much slower moving in N_2_ compared to the degradation in the air. From the TGA curves in the air and nitrogen, we can see that the different trends suggest that the onset of both the first and second phases is highly influenced by oxygen availability.

### 3.4. Self-Healing Efficiency of the Epoxy Samples

#### Evaluation of the Relative Elastic Modulus versus Time of the Epoxy Samples

**Figure 8:**
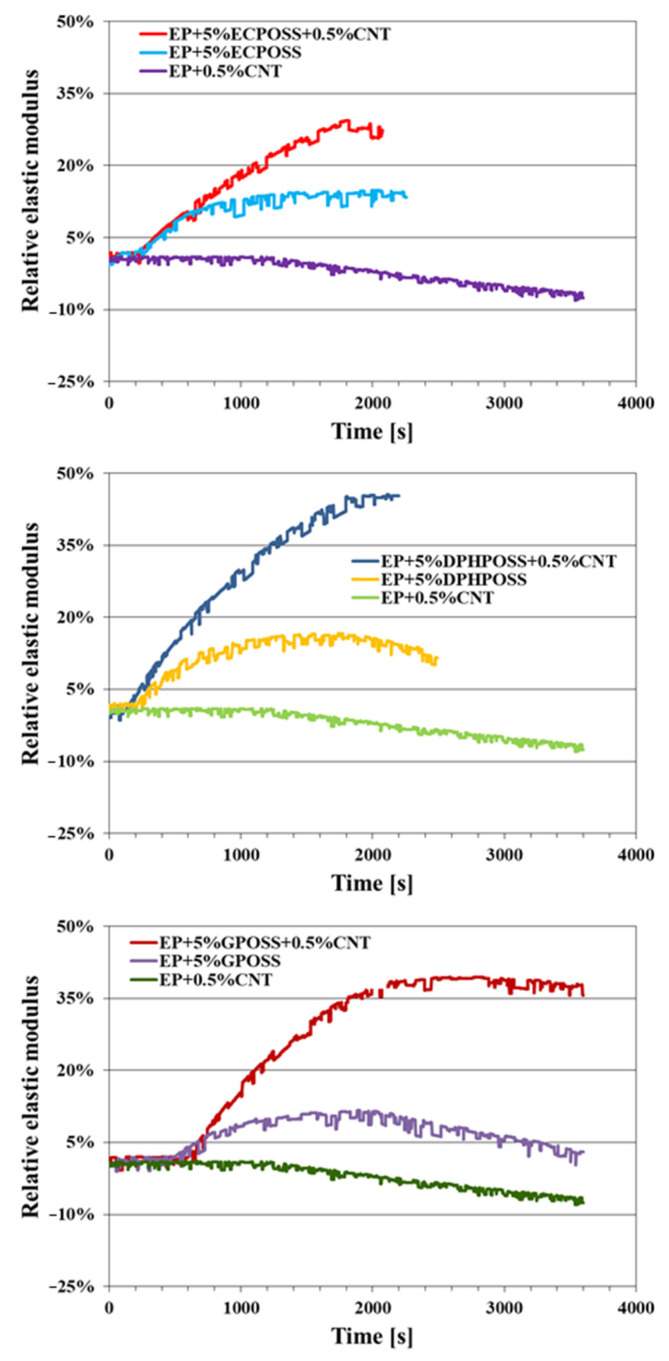
Curves of the relative elastic modulus versus time of the cured epoxy samples: EP+5%ECPOSS+0.5%CNT, EP+5%ECPOSS, EP+0.5%CNT (see graph on the **top**); EP+5%DPHPOSS+0.5%CNT, EP+5%DPHPOSS, EP+0.5%CNT (see graph in the **middle**); EP+5%GPOSS+0.5%CNT, EP+5%GPOSS, EP+0.5%CNT (see graph on the **bottom**).

By evaluating how the relative elastic modulus *G_r_* evolves over time, it is possible to obtain healing efficiency. *G_r_* is defined as follows:

$$G_{r}=\frac{G-G_{c}}{G_{p}-G_{c}}$$

where *G* is the real elastic modulus, *G_c_* is the elastic modulus after the crack has formed, and *G_p_* is the elastic modulus of the sample before the crack has formed. Below, for the three multifunctional nanocomposites, the values of the healing efficiency, expressed as the percentage of elastic modulus recovered, are shown. Each of them is compared with the sample in which only the POSS molecule is present and with the sample in which only the carbon nanotubes are present. In particular, Figure 8 shows the curves of the relative elastic modulus versus time of the following epoxy samples: EP+5%ECPOSS+0.5%CNT, EP+5%ECPOSS, EP+0.5%CNT (see graph on the top); EP+5%DPHPOSS+0.5%CNT, EP+5%DPHPOSS, EP+0.5%CNT (see graph in the middle); EP+5%GPOSS+0.5%CNT, EP+5%GPOSS, EP+0.5%CNT (see graph on the bottom).

From Figure 8, we can observe that, for the sample EP+0.5%CNT, no self-healing efficiency was measured, indicating that the carbon nanotubes are not capable of conferring self-healing functionality to the epoxy resin.

Unlike carbon nanotubes, however, POSS compounds are capable of imparting self-healing functionality to the epoxy resin since, for both sample EP+5%ECPOSS and sample EP+5%DPHPOSS, a self-healing efficiency equal to 15% was recorded while for the EP+5%GPOSS sample, a self-healing efficiency is equal to 10%.

For multifunctional nanocomposites EP+5%ECPOSS+0.5%CNT, EP+5%DPHPOSS+0.5%CNT, EP+5%GPOSS+0.5%CNT, the highest self-healing efficiency values of 30%, 45%, 40% were recorded, respectively. They are due to the combination of carbon nanotubes with POSS compounds, between which a synergistic effect is exerted due to the activation of reversible hydrogen bonds within the resin. The activation of these intermolecular attractive forces is made possible thanks to the structure and composition of the nanoscale POSS cages, which have an inorganic core and organic functional groups capable of hydrogen bonding.

### 3.5. FESEM Morphological Investigation of the Epoxy Samples

#### Evaluation of the Nanofiller Dispersion and the Nanofiller–Epoxy Matrix Interfacial Interactions

**Figure 9:**
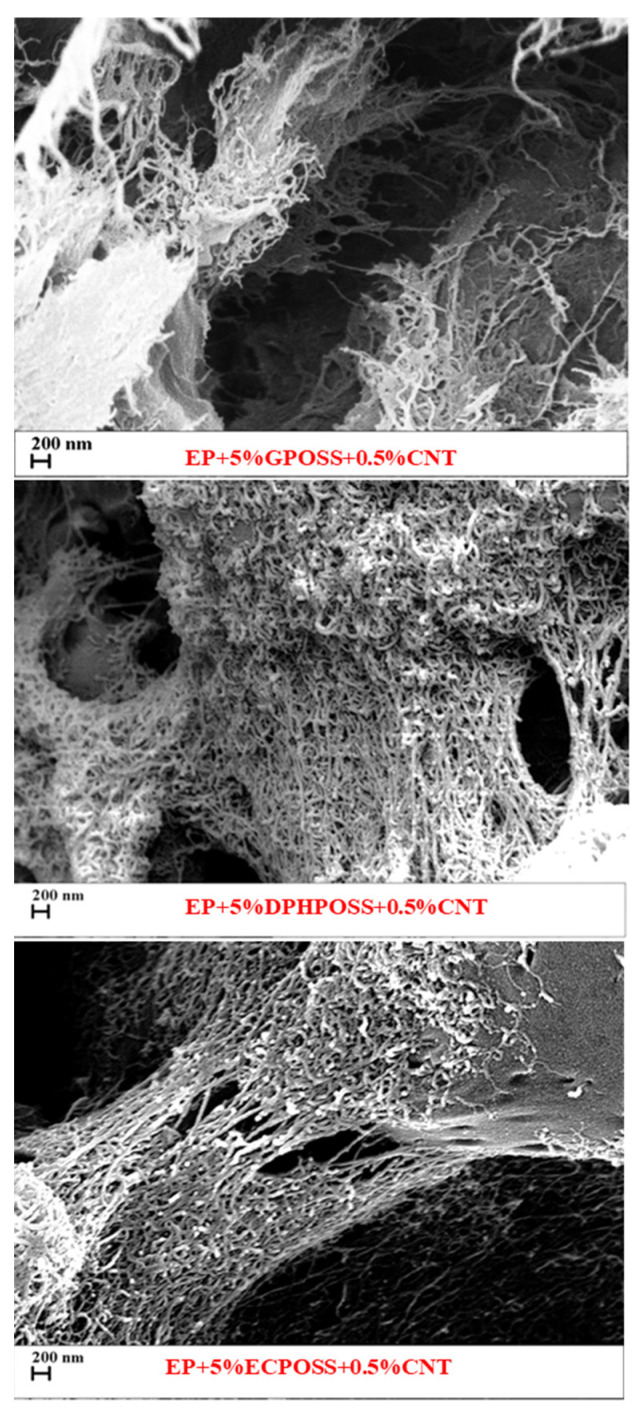
FESEM images of the etched surfaces of the multifunctional nanocomposites.

In order to evaluate the dispersion state of the carbon nanotubes and the nanofiller–epoxy matrix interfacial interactions, a morphological analysis of the fracture surfaces of the multifunctional nanocomposites was performed by FESEM. Figure 9 shows FESEM images with the same scan size (200 nm) of the etched surfaces of the multifunctional nanocomposites. The presence of carbon nanotubes is evident, appearing well-distributed within the matrix to form a network with good interpenetration between them. Furthermore, the etching procedure to which the samples have been subjected and described in Supplementary Materials, causing the consumption of the amorphous resin, allows stripping the carbon nanotubes, which are effectively separated from each other thanks to sonication and are organized in such a way as to create the connections with various sample areas. The existence of intense interactions between the epoxy matrix and POSS also favors a good dispersion of CNTs, which explains the good thermal, mechanical, and electrical performances exhibited by multifunctional nanohybrids.

### 3.6. TUNA Morphological Investigation of the Epoxy Samples

#### Mapping of the Conductive Nanofiller Distribution and Local Electrical Properties of the Nanometric Domains

**Figure 10:**
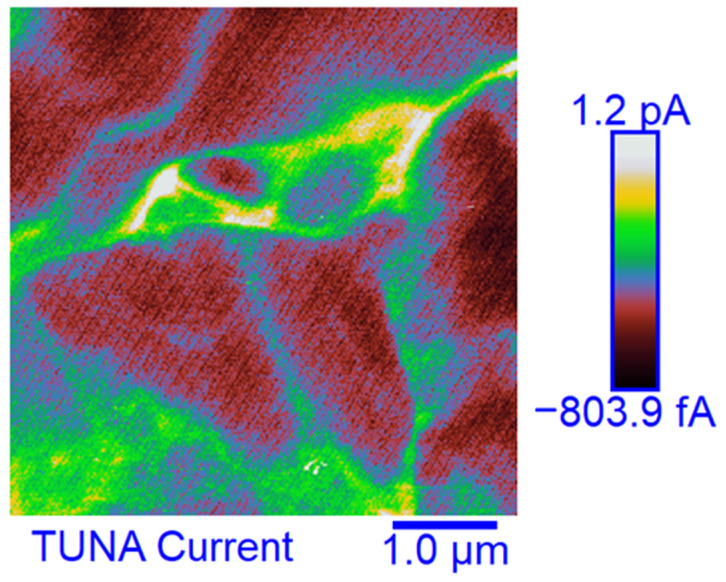
TUNA Current image of the etched surface of EP+5%GPOSS+0.5%CNT.

**Figure 11:**
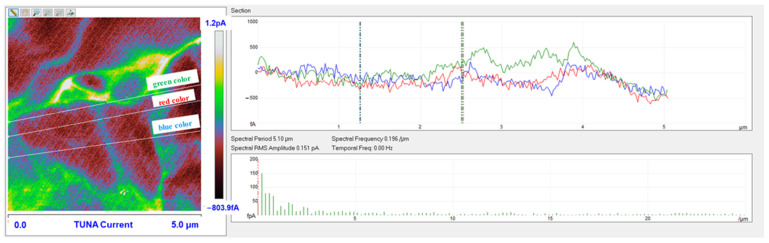
Profile of the current variations of the etched surface of EP+5%GPOSS+0.5%CNT.

**Figure 12:**
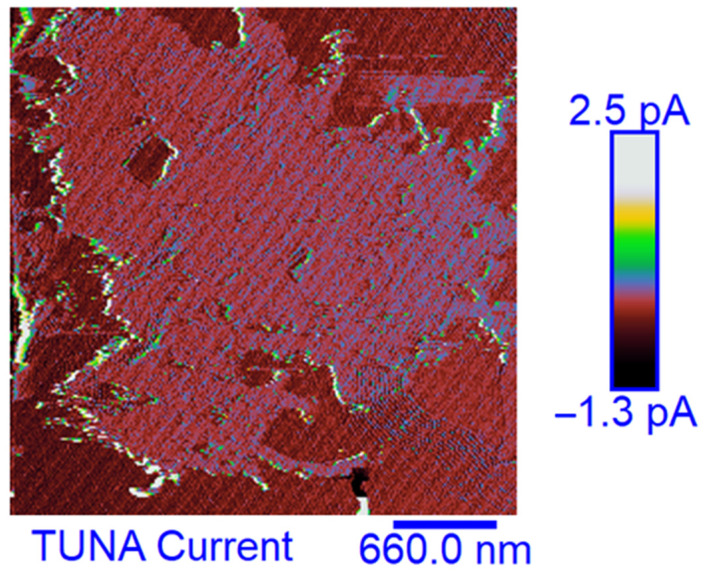
TUNA Current image of the etched surface of EP+5%DPHPOSS+0.5%CNT.

**Figure 13:**
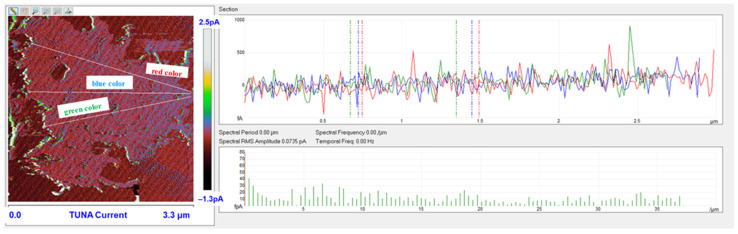
Profile of the current variations of the etched surfaces of EP+5%DPHPOSS+0.5%CNT.

**Figure 14:**
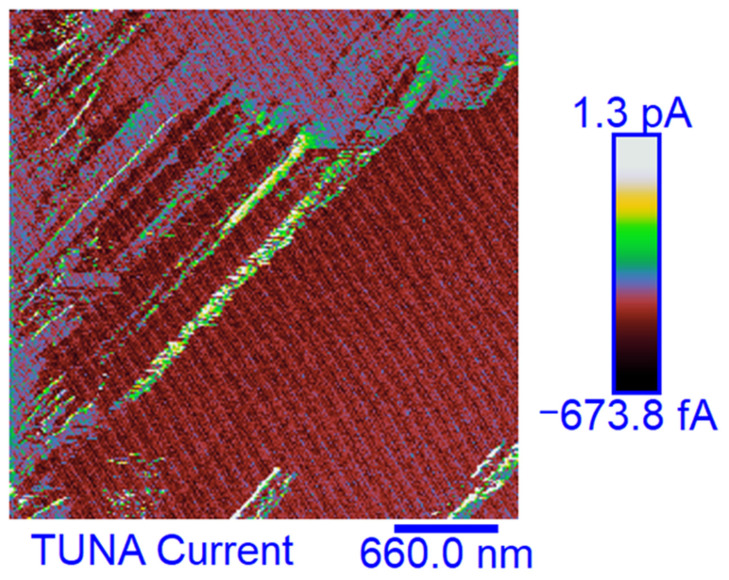
TUNA Current image of the etched surface of EP+5%ECPOSS+0.5%CNT.

**Figure 15:**
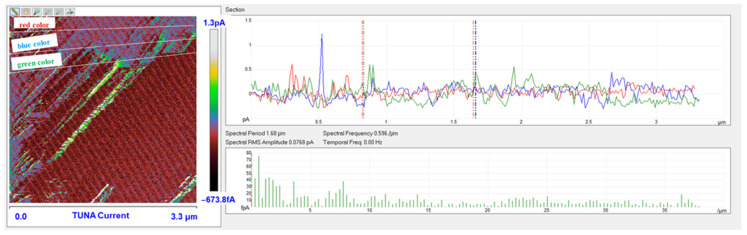
Profile of the current variations of the etched surfaces of EP+5%ECPOSS+0.5%CNT.

Morphological characterization was performed by the TUNA technique to obtain a mapping of the distribution of the conductive nanocharge inside the epoxy matrix and its influence on the local electrical properties of the nanometric domains of the multifunctional nanocomposites. This characterization highlighted the presence of strong interconnections between the carbon nanotubes and the matrix, thus allowing to obtain an effective correlation with the properties of the nanocomposites evaluated with other techniques. The samples were analyzed as they are, without carrying out any preventive treatment aimed at obtaining an electrical connection with the ground. The TUNA analysis also allowed us to obtain information on the dispersion state of the carbon nanotubes. In this work, we report the TUNA Current images of the multifunctional nanohybrids where the chosen nanofiller amount of 0.5 wt% is above the Electrical Percolation Threshold (EPT) [4,30,69,70]. For all three samples, the bulk electrical conductivity values of the order of 10^−3^ S/m, which are consistent with the electric current values measured by the TUNA analysis, were measured. Figure 10 and Figure 11 show the TUNA Current image and profile of the current variations of the etched surfaces of EP+5%GPOSS+0.5%CNT, respectively. Figure 12 and Figure 13 show the TUNA Current image and profile of the current variations of the etched surfaces of EP+5%DPHPOSS+0.5%CNT, respectively. Figure 14 and Figure 15 show the TUNA Current image and profile of the current variations of the etched surfaces of EP+5%ECPOSS+0.5%CNT, respectively. The homogeneous distribution of the carbon nanotubes within the matrix for the three samples is visible. On the right of each TUNA Current image, we can see the scale bar where it is possible to read the electric current values associated with a range of colors with different shades ranging from dark burgundy to light burgundy, then passing through light blue, green, yellow, and light grey. The electric current values increase from the darkest to the lightest color that is reproduced in the topographical image. From the TUNA images, we can easily discriminate the presence of carbon nanotubes that appear on the surface with a lighter color and the strong interconnections due to hydrogen bonds that are established between them and with the epoxy matrix by creating bridges through which an effective transfer of electric nanocharge takes place at the interface with the matrix. The ability of carbon nanotubes to act synergistically with the POSS compounds can be associated with the fact that a continuous conductive network connects different areas of the samples, thus allowing to measure high electric-current values in the nanodomains present on the scanned surface. In particular, for sample EP+5%GPOSS+0.5%CNT, the electric current values range from −803.9 fA to 1.2 pA (Figure 10), for sample EP+5%DPHPOSS+0.5%CNT, the values of electric current range from −1.3 pA to 2.5 pA (Figure 12), and for sample EP+5%ECPOSS+0.5%CNT, the values of electric current range from −673.8 fA to 1.3 pA (Figure 14). From TUNA Current images, we evaluated the dispersion state of the carbon nanotubes in the epoxy matrix. In this regard, Figure 11, Figure 13, and Figure 15 show the profile of the current variations (see green, red, and blue colors on the right picture) correlated with the TUNA current images (see the three white lines on the left picture) of the etched surface of EP+5%GPOSS+0.5%CNT, EP+5%DPHPOSS+0.5%CNT, and EP+5%ECPOSS+0.5%CNT, respectively. It is worth noting that the filler/matrix interchanges along the three white lines in the TUNA Current pictures give the frequency of the current variations. It can easily be seen how the carbon nanotubes are well distributed within the matrix by observing how the frequency of the current variations is somewhat constant for the three multifunctional nanocomposites. Furthermore, the similar trend regarding the intensity of the current variations shown by the three samples is in line with the similar values of electrical conductivity.

## 4. Conclusions

In this work, we demonstrated that multifunctional materials had been successfully designed to manifest synergistic effects between the different functionalities imparted by the effective combination of the carbon nanotubes with the nanostructured POSS cages with the formation of strong intermolecular attractive interactions based on hydrogen bonding. This synergy, through a targeted choice of nanostructured materials and a fine balancing of the properties conferred by every single nanofiller, has led to a simultaneous improvement in the thermal, mechanical, and electrical performance of the multifunctional nanocomposites compared to both the unfilled epoxy matrix and the matrix loaded with carbon nanotubes. More specifically, POSS-based samples turned out to be excellent candidates for aeronautic and aerospace applications thanks to the formation of a highly crosslinked 3D network characterized by high thermal stability, high Tg, elastic modulus, good self-healing properties, and excellent intrinsic electrical conductivity at the nanometer level. The main results are summarized below:

- Dynamic mechanical analysis (DMA) shows that, among the multifunctional nanocomposites, the highest storage modulus values over the whole investigated temperature range were recorded for the sample EP+5%GPOSS+0.5%CNT, loaded with CNTs and GPOSS, which is molecularly solubilized in the hosting liquid epoxy precursors due to the presence of oxirane rings in GPOSS structure, thus creating a continuous crosslinking network in the epoxy matrix; this nanocomposite manifests a trend of the storage modulus similar to that of the sample EP+0.5%CNT which contains only the CNTs;
- All multifunctional formulations have a Tg centered at values close to 260 °C, which fully satisfy structural requirements;
- The calorimetric data well explain the mechanical behavior of the analyzed samples;
- For all three multifunctional nanohybrids, the bulk electrical conductivity values of 10^−3^ S/m are consistent with the electric current values measured by the TUNA analysis;
- For the samples EP+5%GPOSS+0.5%CNT and EP+5%DPHPOSS+0.5%CNT, a thermostability similar to that shown by the unfilled resin EP was observed, whereas an increase in thermostability with respect to the EP resin was observed for the EP+0.5%CNT and EP+5%ECPOSS+0.5%CNT samples, especially in the second stage of the degradation process;
- The combined action of POSS with CNTs has allowed obtaining the highest values of self-healing efficiency (30%, 45%, 40% for EP+5%ECPOSS+0.5%CNT, EP+5%DPHPOSS+0.5%CNT, EP+5%GPOSS+0.5%CNT, respectively) if compared to those measured for samples containing only ECPOSS, GPOSS, DPHPOSS in the absence of CNTs (15% for EP+5%ECPOSS and EP+5%DPHPOSS, 10% for EP+5%GPOSS);
- The sample EP+0.5%CNT containing only carbon nanotubes shows no self-healing efficiency.

## Figures and Tables

**Table 1:** Values of total heat of reaction (ΔH_T_), residual heat of reaction (ΔH_Res_), and DC for the epoxy samples.

	ΔH_T_	ΔH_Res_	DC
	[J/g]	[J/g]	[%]
EP	659.29	46.15	93
EP+0.5%CNT	571.91	62.91	89
EP+5%DPHPOSS+0.5%CNT	479.11	28.21	94
EP+5%ECPOSS+0.5%CNT	510.09	71.56	86
EP+5%GPOSS+0.5%CNT	568.59	51.17	91

**Table 2:** Values of thermal degradation temperatures T_d5%_ and T_d10%_ in Air and Nitrogen flow and the residue at 900 °C.

	Air Flow			Nitrogen Flow		
	T_d5%_	T_d10%_	Residue at 900 °C	T_d5%_	T_d10%_	Residue at 900 °C
EP	358.94	373.07	1.44	349.37	369.69	0.7009
EP+0.5%CNT	362.94	373.94	3.97	360.21	372.34	28.11
EP+5%DPHPOSS +0.5%CNT	363.12	376.67	4.31	365.62	378.22	28.57
EP+5%ECPOSS +0.5%CNT	366.23	377.55	4.15	366.70	376.698	28.02
EP+5%GPOSS +0.5%CNT	359.60	373.36	3.65	359.83	374.032	20.24

## Data Availability

Not Applicable.

## Supplementary Materials

The following supporting information can be downloaded at: https://www.mdpi.com/article/10.3390/polym15102297/s1, Figure S1: Chemical structures of GPOSS, ECPOSS, DPHPOSS; Figure S2: Test geometry adapted for the healing tests (see on the left); epoxy specimens before and after healing experiments (see on the right).
