# Supplementary material for: Multifunctional Properties of Polyhedral Oligomeric Silsesquioxanes (POSS)-Based Epoxy Nanocomposites
Source: Polymers (Basel). 2023 May 13;15(10):2297. doi: 10.3390/polym15102297 (PMC10222506; doi:10.3390/polym15102297)
Supplement: Supplementary file 1 [file polymers-15-02297-s001.zip › polymers-2378028-supplementary.pdf]

Supplementary electronic materials

Multifunctional properties of polyhedral oligomeric silsesquioxanes (POSS)-based epoxy nanocomposites

Liberata Guadagno[1], Andrea Sorrentino[2], Raffaele Longo[1] and Marialuigia Raimondo[1,*]

[1] Department of Industrial Engineering, University of Salerno, Via Giovanni Paolo II, 132, 84084, Fisciano, Italy; lguadagno@unisa.it (L.G.); rlongo@unisa.it (R.L.)
[2] Institute for Polymers, Composites, and Biomaterials (IPCB-CNR), via Previati n. 1/E, 23900 Lecco, Italy; andrea.sorrentino@cnr.it
* Correspondence: mraimondo@unisa.it; Tel: +39-089-964019

Experimental section

The outer diameter and the length of the MWCNTs range from 10 to 30 and from 100 to 1000 nm, respectively. Brunauer–Emmett–Teller method has allowed us to determine the specific surface area of MWCNTs whose value is about 250–300 m^2/g. The thermogravimetric analysis showed a carbon purity greater than 95%.

The chemical structures of GPOSS, ECPOSS, DPHPOSS are shown in Figure S1.

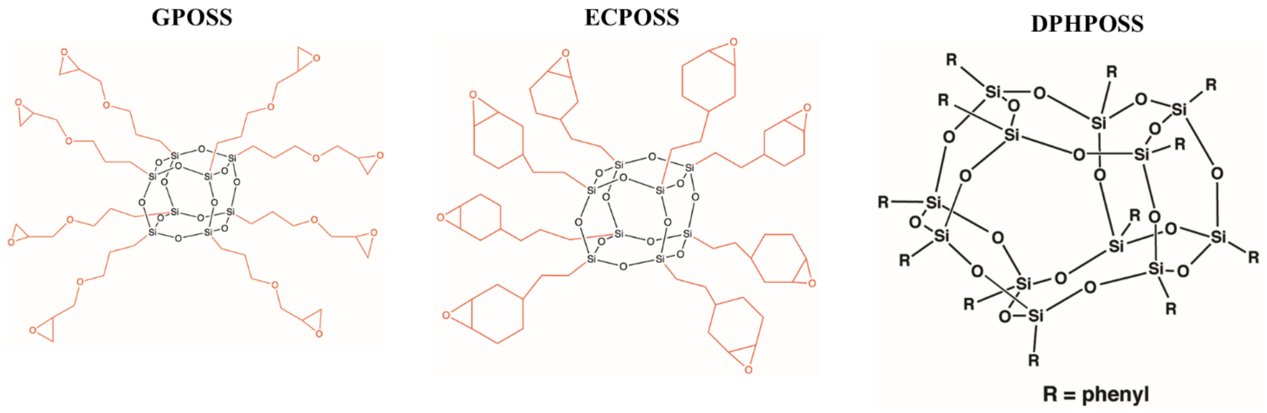

Figure S1. Chemical structures of GPOSS, ECPOSS, DPHPOSS.

The characteristics and aspect of the DPHPOSS, ECPOSS and GPOSS are listed below:

DPHPOSS

(1) Appearance: powder

(2) Color: white

(3) Molecular/chemical formula: $C_{72}H_{60}O_{18}Si_{12}$

(4) Molecular weight: 1550.26 FW

ECPOSS

(1) Appearance: semi-solid

(2) Color: light yellow

(3) Molecular/chemical formula: $(C_8H_{13}O)_n(SiO_{1.5})_n$n = 8, 10, 12

(4) Molecular weight: 1418.20 (for n = 8)

GPOSS

(1) Appearance: viscous liquid

(2) Color: colorless to slightly yellow

(3) Molecular/chemical formula: $(C_6H_{11}O_2)_n(SiO_{1.5})_n$ n = 8, 10, 12

(4) Molecular weight: 1337.88 (for n = 8)

The three different POSS compounds were dispersed in the epoxy blend (TGMDA+BDE) at 5 wt% using two steps consisting of ultrasonication (Hielscher model UP200S (200 W, 24 kHz)) and subsequent magnetic stirring for 1 h in heated oil bath at 120 °C which has a beneficial effect on the quality of the POSS dispersion in the liquid resin. In particular, the stirring step determines: a) the complete dissolution (at molecular level) of the filamentous residues of the GPOSS until the mixture becomes clear since GPOSS being fully epoxidized with glycidyl groups is compatible with epoxy precursors, b) the further reduction of the dimension of the ECPOSS aggregates improving the dispersion of ECPOSS which appears as a random few aggregates; c) any relevant differences in the DPHPOSS dispersion because DPHPOSS is not epoxy-soluble and only the first step must be applied to ensure a better dispersion of DPHPOSS powder in the liquid resins. It is worth noting that the structure of POSS has been shown to have an important role on the dissolution/dispersion of these compound into the matrix. Finally, the multifunctional nanocomposites EP+5%GPOSS+0.5%CNT, EP+5%DPHPOSS+0.5%CNT, EP+5%ECPOSS+0.5%CNT were obtained by adding CNT nanofiller at 0.5 wt% into the liquid mixture containing TGMDA, BDE and POSS compounds through an ultrasonication for 20 min and, then, by solubilizing a stoichiometric quantity of DDS at 120°C for 20 min. The concentration of 0.5 wt% of CNT has been selected because for this concentration, the sample is beyond the electrical percolation threshold (EPT). Furthermore, the sample EP+0.5%CNT was obtained by dispersing CNT at 0.5 wt% within the liquid mixture containing TGMDA and BDE through an ultrasonication for 20 min and, then, by solubilizing a stoichiometric amount of DDS at 120°C for 20 min.

Dynamic mechanical properties of the solid samples with dimensions 2 × 10 × 35 mm^3 were performed with a dynamic mechanical thermo-analyzer (Tritec 2000 DMA -Triton Technology) by applying a variable flexural deformation in three points bending mode. The displacement amplitude was set to 0.03 mm, whereas the measurements were performed at the frequency of 1 Hz. The range of temperature was from 30 °C to 310 °C at the scanning rate of 3 °C/min.

A Bruker Vertex 70 FTIR-spectrophotometer (Bruker Optics Inc. Billerica, MA, USA) allowed to carry out Fourier-transform infrared spectroscopy (FTIR) analysis in the range 4000–400 cm^{-1}, with a resolution of 2 cm-1 (32 scans collected). The infrared spectra of the unfilled and nanofilled resins were acquired in absorbance by spreading the liquid mixture on the KBr pellets.

Two types of thermal investigation were performed on the formulated samples, Differential Scanning Calorimetry (DSC) and Thermogravimetric Analyses (TGA). DSC analyses have been carried out by using a thermal analyser Mettler DSC 822/400 (Mettler-Toledo Columbus, OH, USA) equipped with DSC cell purged with nitrogen and chilled with liquid nitrogen for sub-ambient measurements. DSC has been employed to

evaluate the samples' curing degree (DC), assuming that exothermic heat developed during the curing process is proportional to the extent of the curing reactions. The DC can be determined from the total heat of reaction (ΔH_T) of the curing reactions and the residual heat of reaction (ΔH_{Res}) of the partially cured resin according to Equation 1.

$$DC = \frac{\Delta H_T - \Delta H_{Res}}{\Delta H_T} \times 100 \qquad (1)$$

The total heat of reaction (ΔH_T) has been determined by performing the DSC analysis on the liquid uncured resins, scanning about 7.0 mg of the sample by a heating run at 10 °C/min from 30 to 290 °C, while the ΔH_{Res} was determined from the measurements performed on the oven hardened samples, by scanning the polymerized materials at 10 °C/min from 30 °C to 290 °C.

The analysis of the uncured samples was carried out by means of a dynamic heating program which contemplates three stages in the temperature range between 30 °C and 290 °C, namely: a) a first run from 30 °C up to 290 °C with a scan rate of 10 °C/min, b) a second run from 290 °C to 30 °C with a scan rate of 50 °C/min and c) a third run from 30 °C up to 290 °C with a scan rate of 10 °C/min. The samples oven cured at 200 °C were analyzed by single heating run from 30 °C up to 290°C with a scan rate of 10°C/min.

Thermogravimetric analysis (TGA) (Mettler TGA/SDTA 851 thermobalance) was accomplished by heating the samples from 0°C to 1000°C at a 10°C/min heating rate under both nitrogen and air flows.

Micrographs of the epoxy nanocomposites were obtained using Scanning Electron Microscope-SEM (mod. LEO 1525, Carl Zeiss SMT AG, Oberkochen, Germany). All samples were placed on a carbon tab previously stuck to an aluminum stub (Agar Scientific, Stansted, UK) and were covered with a 250 Å-thick gold film using a sputter coater (Agar mod. 108 A). Nanofilled sample sections were cut from solid samples by a sledge microtome. These slices were etched before the observation by FESEM. The etching reagent was prepared by stirring 1.0 g potassium permanganate in a solution mixture of 95 mL sulfuric acid (95–97%) and 48 mL orthophosphoric acid (85%). The filled resins were immersed into the fresh etching reagent at room temperature and held under agitation for 36 h. Subsequent washings were done using a cold mixture of two parts by volume of concentrated sulfuric acid and seven parts of water. Afterward the samples were washed again with 30% aqueous hydrogen peroxide to remove any manganese dioxide. The samples were finally washed with distilled water and kept under vacuum for 5 days before being subjected to morphological analysis.

To assess the crack-healing efficiency of the composite materials, experiments were performed by using a dynamic mechanical thermo-analyzer (TA instrument-DMA 2980). Solid samples with dimensions 3 × 10 × 35 mm^3 were tested by applying a variable flexural deformation in single cantilever mode. A V-shaped starter notch, 1 mm deep and 2 mm wide, was machined close to the sample extremity as shown in Figure S2.

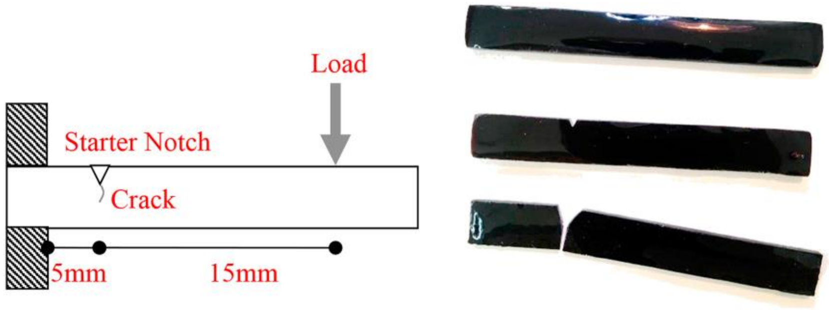

Figure S2. Test geometry adapted for the healing tests (see on the left); epoxy specimens before and after healing experiments (see on the right).

The test procedure combines three different steps. In the first step, the notched sample was analyzed under the dynamic flexural deformations for about 300 s in order to determine the pristine elastic modulus. After that, the oscillation was stopped and a sharp pre-crack was created in the samples by gently tapping a fresh razor blade into a machined starter notch. An impulsive load of about 25 N was immediately applied to the specimen in order to produce crack propagation along the virgin crack plane. At this point, the dynamic monitoring of the sample was conducted at constant temperature, with a displacement amplitude set to 0.1% and a frequency to 1 Hz. Several test conditions were investigated in order to obtain a reasonable picture of the amount and time of recovery without influencing the healing mechanisms.

Information on topography and local nanoelectrical current of the multifunctional nanocomposites was obtained by TUNA technique operating in contact mode and using platinum-coated probes with nominal spring constants of 35 N m−1 and electrically conductive tip of 20 nm.

The TUNA module measures ultra-low currents (<1 pA) ranging from 80 fA to 120 pA circulating through the conductive tip to the investigated samples kept at a fixed DC bias. In this work, we used a DC sample bias from 1 V to 2 V.

A linear current amplifier with a range of 60-120 fA detects the resulting current passing through the samples. In this way, the sample's topography and current are measured at the same time, activating direct correlation of a sample location with its electrical properties.

It is worth noting that notably sensitive current measurements are allowed due to the noise level of the TUNA module commonly of 50 fA.

Highest resolution current mapping of the nanocomposites was obtained with the current sensitivity of the TUNA module which selects the gain referring to the output voltage of the TUNA sensor set to 1pA/V, corresponding to the gain of 10 12 , scan rate of 0.500 Hz s^{-1}, number of pixels in X and Y (samples/line) set to 512.

The detected areas of the analyzed samples are representative of the entire multifunctional nanocomposites because, in order to obtain electrical measurements at nanoscale level with indisputable repeatability and reproducibility, a cantilever with a sharp tip has scanned on different areas over a sample surface so that each TUNA image reported in the manuscript was captured after verifying that the electrical response was the same at least five various scanned points.

It is worth noting that, generally, it is not enough that the tip is in contact with a conductive material but electrical contacts to the ground ensured by silver paste are also essential for the current to flow.

Thus, a current signal is obtained only if the tip during the sample contact constitutes a part of a closed electrical circuit. In this work, the nanoelectrical characterization was carried out without grounding the samples.

The TUNA results show that, even if the analyzed samples are not grounded, it is possible to detect electric current values that irrefutably prove the intrinsic electrical conductivity of the formulated nanocomposites.

The TUNA images were analyzed using the Bruker software Nanoscope Analysis 1.80 (Build R1.126200). To highlight the morphological peculiarities of the samples as well as their distribution within the polymeric matrix and their affinity with the epoxy domains, the nanocomposites underwent etching treatment before being investigated by TUNA. It is worth noting that thanks to the possibility of detecting currents and, at the same time, providing details on the fine morphological characteristics of the conductive nanocharge inside the host matrix, TUNA technique gives helpful further information of the nanoscale material properties.
